# Bystander effects of ionizing radiation: conditioned media from X-ray irradiated MCF-7 cells increases the angiogenic ability of endothelial cells

**DOI:** 10.1186/s12964-019-0474-8

**Published:** 2019-12-16

**Authors:** Nasrollah Jabbari, Muhammad Nawaz, Jafar Rezaie

**Affiliations:** 1 Solid Tumor Research Center, Cellular and Molecular Medicine Institute, Urmia University of Medical Sciences, Urmia, Iran; 2 Department of Rheumatology and Inflammation Research, Institute of Medicine, Sahlgrenska Academy, University of Gothenburg, Gothenburg, Sweden

**Keywords:** Bystander effects, Angiogenesis, Endothelial cells, Breast cancer, Radiotherapy

## Abstract

**Background:**

Non-targeting effects of radiotherapy have become as clinical concern due to secondary tumorigenesis in the patients receiving radiotherapy. Radiotherapy also affects non-tumoral cells present in the tumor microenvironment and surrounding tissues. As such, the irradiated cells are thought to communicate the signals that promote secondary tumorigenesis by affecting the function and fate of non-irradiated cells in the vicinity including endothelial cells. This may include up-regulation of genes in irradiated cells, secretion of paracrine factors and induction of gene expression in surrounding non-irradiated cells, which favor cell survival and secondary tumorigenesis. In the current study, we aimed to investigate whether the conditioned media from X-ray irradiated MCF-7 cells contribute to induction of gene expression in human umbilical vein endothelial cells (HUVECs) in vitro and modulate their angiogenic capability and migration.

**Methods:**

Following the co-culturing of X-ray irradiated MCF-7 media with HUVECs, the migration and wound healing rate of HUVECs was monitored using Transwell plate and scratch wound healing assay, respectively. The levels of angiogenic protein i.e. vascular endothelial growth factor (VEGF-A) in the conditioned media of MCF-7 cells was measured using ELISA. Additionally, we quantified mRNA levels of VEGFR-2, HSP-70, Ang-2, and Ang-1 genes in HUVECs by real time-PCR. Tubulogenesis capacity of endothelial cells was measured by growth factor reduced Matrigel matrix, whereas expression of CD34 (a marker of angiogenic tip cells) was detected by flow cytometry.

**Results:**

Data showed that VEGF-A protein content of conditioned media of irradiated MCF-7 cells was increased (*P* < 0.05) with increase in dose. Data showed that irradiated conditioned media from MCF-7 cells, when incubated with HUVECs, significantly enhanced the cell migration and wound healing rate of HUVECs in a dose-dependent manner (*P* < 0.05). The mRNA levels of VEGFR-2, HSP-70, Ang-2, and Ang-1 were dose-dependently enhanced in HUVECs incubated with irradiated conditioned media (*P* < 0.05). Importantly, HUVECs treated with irradiated conditioned media showed a marked increase in the tube formation capability as well as in expression of CD34 marker (*P* < 0.05).

**Conclusions:**

Our findings indicate that conditioned media from irradiated MCF-7 cells induce angiogenic responses in endothelial cells in vitro*,* which could be due to transfer of overexpressed VEGF-A and possibly other factors secreted from irradiated MCF-7 cells to endothelial cells, and induction of intrinsic genes (VEGFR-2, HSP-70, Ang-2, and Ang-1) in endothelial cells.

**Video abstract.**

**Graphical abstract:**

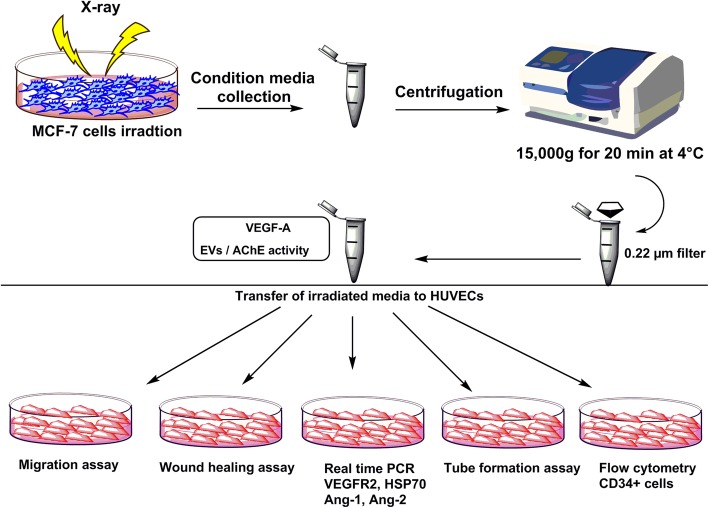

## Background

Breast cancer, which most frequently arises within cells in the milk-making ducts, is a globally occurring malignancy and has become a mortality issue worldwide among women [1]. It has been reported that more than 2 million women were affected by breast cancer in 2018 [2]. Several attempts including, but not limited to, radiotherapy, surgery, chemotherapy, and hormonal therapy are being made to enhance patients’ survival [3, 4]. Radiotherapy, an essential X-ray therapy, also known as ionizing radiation, is frequently applied therapy to eliminate tumors in patients suffering from cancer [5]. Nonetheless, resistance responses, tumor relapse, and metastasis have remained a clinical concern after radiotherapy [6].

Previous studies reported that radiotherapy causes non-targeting effects (NTE) and alteration in tumor cells’ dynamic; whereby irradiated cells mediate the systemic effects of localized radiotherapy to non-irradiated cells located nearby and/or distant sites [6, 7]. Bystander effects (BEs), a subtype of NTE, implies that radiation may indirectly affect non-irradiated cells within an irradiation volume by secreted molecules to disseminate the biological effects of radiation [8]. In this context, BEs can be mediated through different soluble factors such as growth factors, cytokines, reactive oxygen species [8], as well as extracellular vesicles (EVs) and/ or direct junctions between cells e.g., tunneling nanotubes (TnTs) [9, 10]. Bystander cells, which receive signals from irradiated cells, may display the same properties as irradiated cells [11, 12]. In recent years, the investigation of radiation-induced BEs has been exploited to assess responses of non-irradiated cells to signals from irradiated cells [11, 13].

Irradiated tumor cells relay their bystander effects on non-tumoral cells, which promote secondary tumorigenesis in the vicinity [14, 15], including the induction of angiogenesis which favor cell survival and invasion [16].

Angiogenesis, i.e. formation of new blood vessels from pre-existing vascular network, is a critical step in tumor growth as well as metastasis [17]. Undeniably, tumor retention and growth depends on a sufficient source of nutrients and oxygen supply, where formation of neovasculature (angiogenesis) keeps nutritional and oxygen supply to growing tumor volume [18]. It is well established that angiogenesis is a regulated process and various molecules contribute to promote angiogenesis, both during physiological and pathological conditions [17]. Molecules or factors released from irradiated cells may promote tumor growth through inducing angiogenic responses in endothelial cells [19].

Additionally, irradiated cancer cells are capable of releasing angiogenic factors such as VEGF [20] and matrix metalloproteinases (MMPs) [21] into extracellular milieu and cause BEs-mediated carcinogenesis. VEGF-A is a glycosylated mitogen protein and is involved in angiogenesis [22]. Indeed, endogenous expression of VEGF-A or induction by extrinsic stimulators, specifically in endothelial cells, participate in orchestrating several cellular responses including cell proliferation, apoptosis, migration, vascular permeability, and angiogenesis [23, 24].

Beside the secretion of VEGF-A and extracellular vesicles in CM, the ionizing radiation may cause the release of several soluble factors including matrix metalloproteinases (MMPs), urokinase-type plasminogen activator (uPA) [25], epidermal growth factor (EGF) and IL-1β [26], and basic fibroblast growth factor (bFGF) [27]. These factors elicit BEs on recipient cells and activate angiogenic responses in endothelial cells.

In a preclinical model, it was reported that ionizing radiation destroyed tumor vasculature and proliferation, however migration of angiogenic CD11b + mononuclear cells from the bone marrow recovered the vasculature density and induced vasculogenesis in irradiated tumor [28]. Oh et al. found that ionizing radiation inhibited angiogenesis in endothelial cells obtained from tumorous breast tissues, whereas promoted angiogenesis in endothelial cells of normal ones [29]. Parthymou and co-workers demonstrated that conditioned media (CM) from irradiated C6 glioma cells enhance survival and migration of human umbilical vein endothelial cells (HUVECs) in vitro [30]. Moreover, CM from irradiated umbilical cord blood-derived mononuclear cells induced angiogenic factors in HUVECs and endothelial progenitor cells and promoted angiogenesis in vitro [31]. Although, reports have shown that irradiated endothelial cells exhibit angiogenic responses [32, 33], however, bystander effects of irradiated cells on endothelial cells have not been fully known. In current, study we aimed to investigate the contribution of conditioned media from X-ray irradiated MCF-7 human breast cancer cells for possible induction of gene expression in non-irradiated endothelia cells (HUVECs), migration and their angiogenic potential.

## Methods

### Cell culture

Human breast cancer cell (MCF-7) and HUVEC lines were obtained from Pasteur institute (Iran). Cells were cultured in Dulbecco’s Modified Eagle’s Medium (DMEM; Gibco) enriched with 10% FBS (Gibco) and 1% penicillin streptomycin (Gibco). The cells were grown in a CO2 incubator at 37 °C. Every 2–3 days, cell medium was replaced with fresh medium. Cells between 3 and 6 passages were subjected to experiments.

### Irradiation

A medical linear accelerator system (Siemens AG, Germany) was employed to create high-energy X-ray for irradiation. Using a polystyrene with 1.5 cm thickness, the cell culture flasks were placed at the dmax = 1.6 cm of radiation isocenter position and received X-ray at dose rate of 200 MU/min with a 180° gantry angle where machine was calibrated by dose rate of 1 cGy per monitor unit. MCF-7 cells were allocated into five irradiated groups, which received 2, 4, 6, 8, and 10 Gy of X-ray doses. Non-irradiated cells were considered as control group, which followed the same handling procedure without any X-ray irradiation. After receiving X-ray, the cells were transferred to incubator for downstream examination.

### Condition media collection

To collect conditioned media, 1 h prior to irradiation, cell culture media of all groups were discarded and washed three times with phosphate-buffered saline (PBS). Then FBS- free DMEM was added to flasks and cells were irradiated. After 48 h post-incubation, CMs of both irradiated and control cells were harvested and cell debris was removed by centrifugation (15,000 g for 20 min at 4 °C) and 0.22 μm filter membranes. CMs were stored at − 80 °C until further use.

To assay the angiogenic response of HUVEC against MCF-7 cell derived CMs, HUVEC were divided into five groups as; control-CM, 2 Gy-CM, 4 Gy-CM, 6 Gy-CM, 8 Gy-CM, and 10 Gy-CM and were incubated with related CMs for 48 h. Three–repeated independently biological measures were considered for all experiments.

### Migration assay

HUVECs migration rate was measured using Transwell insert cell culture plate with 8-μm pore size (SPL). For this purpose, 2 × 10^4^ HUVEC were suspended in 200 μl of DMEM and seeded on the insert part of plate while lower chamber filled with 700 μl of CMs from MCF-7 considered as chemoattractant medium over 24 h. The migrated cells to CMs were counted in randomly five high power field (HPF).

### Wound healing assay

To investigate wound healing potential of HUVEC, we performed scratch wound healing assay. In brief, 5 × 10^5^ HUVEC cells per well were seeded in 6-well plates and were grown to reach confluent monolayer. Then using a 1000 μl pipette tip, the scratches were created. Cells were washed three times with PBS and treated with CMs of irradiated and control MCF-7 cells over 48 h. The images of migrated cells were recorded using TrueChrome IIS system (China) at 0 h and 48 h. The percentage of wound closure was analyzed with Image J NIH software ver. 1.44p. and reported by following formula: % healing rate = (New surface area - Last surface area)/ New surface area × 100.

### Enzyme-linked immunosorbent assay

To monitor protein level of VEGF-A in CMs of MCF-7 cells, we performed enzyme-linked immunosorbent assay (ELISA) using human VEGF Quantikine PharmPak kit for VEGF-A (R and D systems, USA), according to manufacturer’s instructions. Briefly, 100 μL of CMs were added to 96-well microwells and incubated for 2 h at room temperature. After washing with PBS, 100 μl of biotin-conjugate was added and following washing; 100 μl of streptavidin-HRP was added to each well and kept for 1 h at room temperature. Next, 100 μl of substrate solution was mixed and incubated for 30 min. The reaction was stopped by addition of 100 μl of stop solution. Color density was read at 450 nm using a microwell reader (BioTek).

### Acetylcholinesterase activity assay

The amount of extracellular vesicles in CMs of MCF-7 cells was detected by acetylcholinesterase (AChE) activity using cholinesterase kit (Cat No. BXC080; Iran). Briefly, reagent A (potassium hexacyanoferrate + pyrophosphate) was added to CMs and kept for 5 min at room temperature. Next, reagent B (2-butyrylthio-n,n,n-trimethylethanaminium iodide) was mixed and the absorbance rate were read at 405 nm by three different rest intervals procedure using a microplate reader instrument (BioTek). AChE activity was reported by applying the provided formula: Activity (U/l) = 65,800 × ΔAbs/min.

### Real time PCR

The mRNA levels of Ang-1, Ang-2, VEGFR-2, HSP-70 genes were calculated using quantitative real time PCR (Q-PCR). HUVEC were cultivated with CMs from MCF-7 over 48 h then following trypsinization, the cells were collected. Total RNA was extracted using commercially available RNA isolation kit (Yekta Tajhiz Azma, Iran), according to instructions. The RNA quality (260/280 ratio) and quantity were measured using a Nanodrop system (BioTek). First-strand complementary DNA (cDNA) was constructed using 1000 ng of RNA through a cDNA synthesis kit (Yekta Tajhiz Azma, Iran). Next, an ABI 7500 Real-Time PCR System (Applied Biosystems) and SYBR Green PCR Master Mix (Yekta Tajhiz Azma, Iran) were employed to measure expression levels of VEGFR-2, Ang-1, Ang-2, HSP-70 genes involved in angiogenesis. The mRNA level of GAPDH gene was considered as normalizing control gene. Fold change values were evaluated using the following formula: 2 ^−ΔΔCt^. All primers used in this study are listed in Table 1.

**Table 1:** List of primers

Genes	Sense	Antisense	Product size	Tm
VEGFR-2	CCAGCAAAAGCAGGGAGTCTGT	TGTCTGTGTCATCGGAGTGATATCC	87	60
HSP-70	GCCGAGCATTCTCTGATCCA	AACACTTTCGGCTGTCTCCT	182	60
Angiopoietin-1	GGACAGCAGGAAAACAGAGC	CACAAGCATCAAACCACCAT	129	63
Angiopoietin-2	ATCAGACCACGAGACTTGAAC	TCCATAGCTAGCACCTTCTTTT	145	60
GAPDH	TTGACCTCAACTACATGGTTTACA	GCTCCTGGAAGATGGTGATG	126	59

### Tube formation assay

Growth factor-free Matrigel (corning life sciences, USA) assay was used to determine effect of CMs from MCF-7 cells on tubulogenesis capacity of HUVEC. Briefly, 50 μl of cold liquefied Matrigel loaded into per well of 96 cell culture plate. Then, the plate was maintained at room temperature for 1 h until Matrigel was polymerized. Next, 15,000 CMs-treated HUVECs were suspended in 100 μl of FBS-free medium and were dispensed onto coated wells for 24 h. The in vitro tube networks were visualized using TrueChrome IIS system (China). Tube total length was calculated by Image J NIH software ver. 1.44p.

### Flow cytometry analysis

HUVECs were incubated with the CMs and the percentage of CD34 positive HVECs was monitored over 48 h post-exposure to CMs. At the end of exposure time, HUVECs were collected and following the fixation by 4% paraformaldehyde solution, the cells were blocked using a binding buffer reagent (Cat no: 00–0055-56, eBioscience) for 15 min at room temperature. Next, cells were stained by a CD34-FITC (Can. No: 345801, BD Biosciences) antibody for 20 min at room temperature. The investigation was accomplished by FACSCalibur system and FlowJo software (ver. 7.6.1).

### Statistical analysis

Data are presented as means ±SD of three biological replicates. Results obtained from different experiments were analyzed by the SPSS software (version 25) and One-way Analysis of Variance (ANOVA) and Tukey test while a *P* value less than 0.05 was considered statistically significant. In graphs, the symbolic asterisks as * *P* < 0.05, **P < 0.01, ***P < 0.001, ****P < 0.0001, and *****P < 0.00001 used to show significant difference between groups.

## Results

### Irradiated conditioned media increased the migration rate of HUVEC

**Fig. 1:**
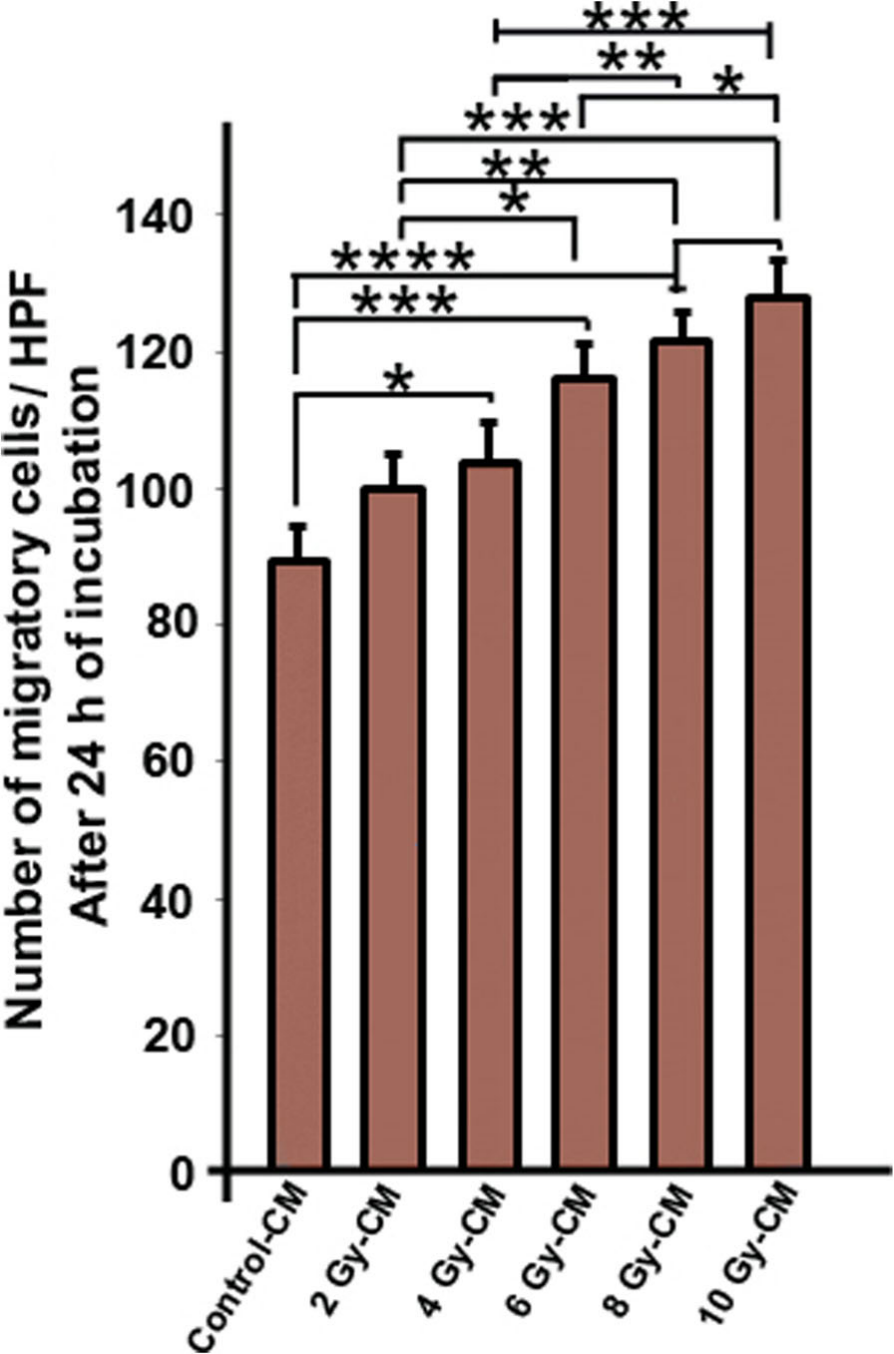
Migration rate of human umbilical vein endothelial cells (HUVECs). Conditioned media from irradiated MCF-7 cells (2Gy to 10 Gy) increased the migration rate of HUVECs after 24 h incubation. One-way ANOVA with Tukey test was used. All values are shown as means ± SD; (3 biologically independent experiments). * P < 0.05, ** *P* < 0.01, *** P < 0.001, **** *P* < 0.0001. HPF means High Power Field

The migration potential of HUVECs toward irradiated conditioned media (CM) was monitored after 24 h using a Transwell plate. In comparison to control group, the migration rate of endothelia cells was significantly increased when irradiated CMs were used as chemoattractant (*P*_*Control* vs.*4 Gy-CM group*_ *<* 0.05; *P*_*Control* vs.*6 Gy-CM group*_ *<* 0.001; *P*_*Control* vs. *8 Gy-CM and 10 Gy-CM group*_ *<* 0.0001) (Fig. 1). The same results were observed when 2 Gy-CM group was compared with other Gy groups (*P*_*2Gy-CM* vs.*6 Gy-CM group*_ *<* 0.05; *P*_*2Gy-CM* vs.*8 Gy-CM group*_ *<* 0.01; *P*_*2Gy-CM* vs. *10 Gy-CM group*_ *<* 0.001). Compared to 4Gy-CM group, an increased level of migrated cells in 8 Gy-CM group and 10 Gy-CM group was observed (*P* < 0.01 and *P* < 0.001 respectively). In addition, a significant difference between 6 Gy-CM and 10 Gy-CM was observed (*P <* 0.01) (Fig. 1). The data show the HUVEC migration rate dose-dependently increased towards CMs with high doses of X-ray in irradiated MCF-7 cells, after 24 h. This indicates that secreted factors in radiated CMs act as chemoattractant which may stimulate migration of HUVECs with raise in radiation dose.

### Irradiated conditioned media enhanced the wound healing rate

**Fig. 2:**
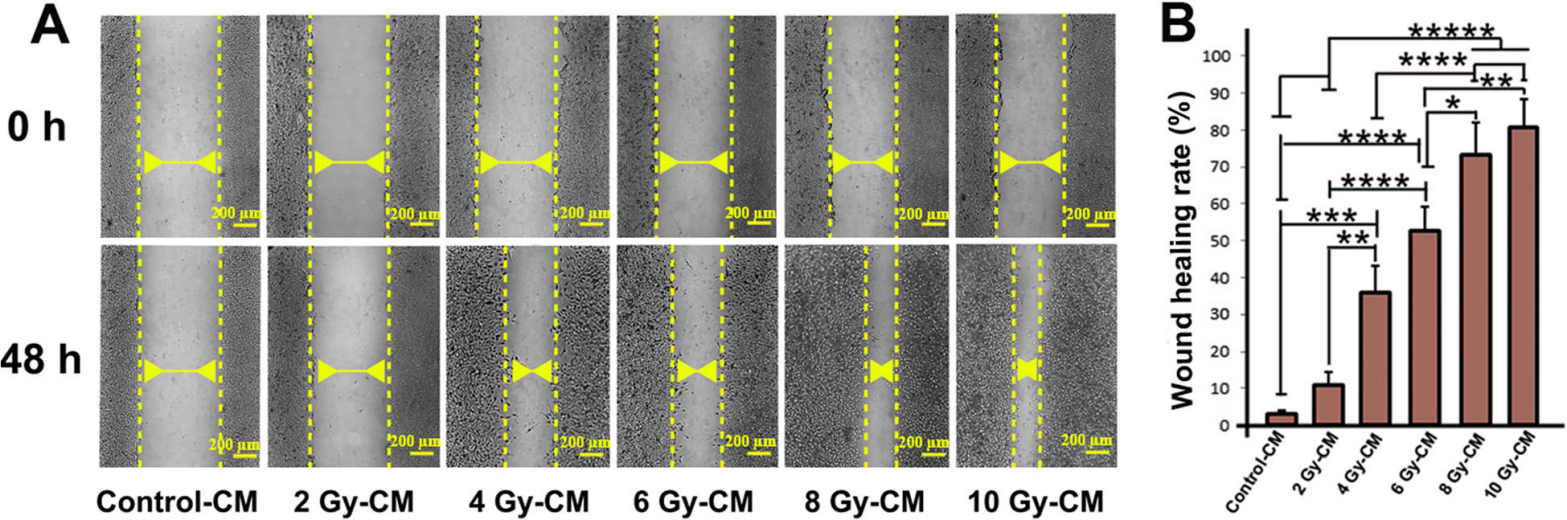
The wound healing rate of HUVECs evaluated by in vitro scratch assay over a period of 48 h incubation of irradiated media with HUVECs (**a**, **b**). (Scale bar: 200 μm). One-way ANOVA with Tukey test was used. All values are shown as means ± SD; (3 biologically independent experiments). * P < 0.05, ** *P* < 0.01, *** P < 0.001, **** *P* < 0.0001, ***** *P* < 0.00001

For further examination, we measured migration capacity of HUVECs by in vitro scratch wound healing assay. Data showed that the percentage of wound healing rate dose-dependently increased when cells were incubated with irradiated CMs (*P*_*Control-cm* vs. *4Gy-CM group*_ *<* 0.001; *P*_*Control-CM* vs.*6 Gy-CM group*_ *<* 0.0001; *P*_*Control-CM* vs. *8 Gy-CM and 10 Gy-CM group*_ *<* 0.00001) (Fig. 2a, b). The similar increasing trend in wound healing rate was observed when 2 Gy-CM group compared to other groups (*P*_*2Gy-cm* vs. *4Gy-CM group*_ *<* 0.01; *P*_*2Gy-CM* vs.*6 Gy-CM group*_ *<* 0.0001; *P*_*2Gy -CM* vs. *8 Gy-CM and 10 Gy-CM group*_ *<* 0.00001). In comparison to 4 Gy-CM group, the wound healing rate of endothelial cells was significantly increased in 8 Gy-CM group and 10 Gy-CM group (*P* < 0.0001). In addition, we found that the wound healing rate of 8 Gy-CM group (73.3 + 8.6%) and 10 Gy-CM (80.7 ± 7.8%) group was significantly increased in comparison to 6 Gy-CM group at end-stage of the test (52.76 ± 6.63%; *P* < 0.05 and *P* < 0.01 respectively)(Fig. 2a, b). These results indicate that CMs of irradiated MCF-7 cells may contain secreted factors, which activate reparative mechanisms (proliferation and wound healing) of HUVECs, in dose dependent manner.

### Ionizing radiation increased VEGF-A secretion into supernatants of MCF-7 cells

After having observed the contribution of irradiated media (but unknown factors) on migration and wound healing in HUVECs, we wanted to investigate which secreted factors in CM are responsible for these functions. To examine this, we measured the amount of secreted VEGF-A protein in supernatants of irradiated MCF-7 cells. VEGF-A is a glycosylated mitogen protein, which promotes cell proliferation, migration, vascular permeability, angiogenesis in endothelial cells [29, 30].

**Fig. 3:**
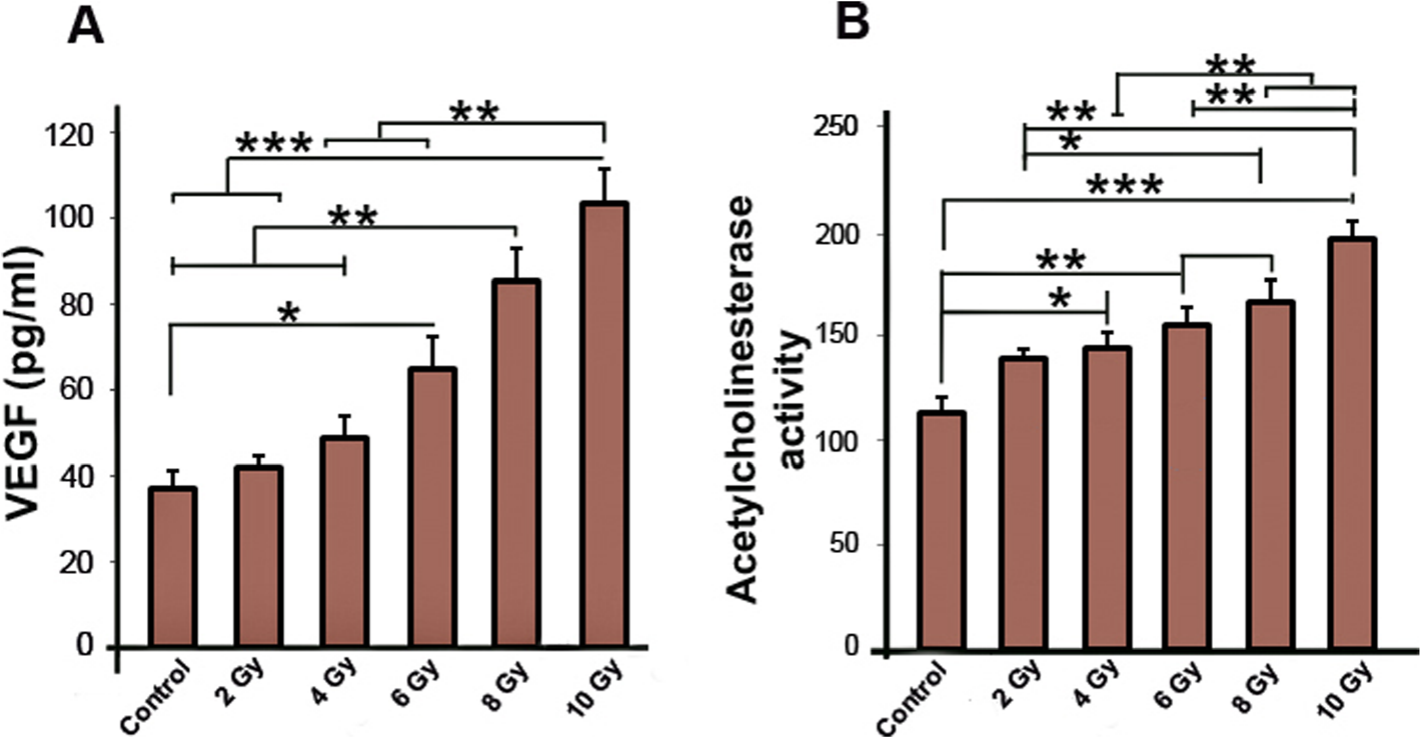
Eenzyme-Llinked Iimmunosorbent Aassay (ELISA) of VEGF-A protein in conditioned media of MCF-7 cells. Data show that the amount of VEGF-A was increased in conditioned media of the irradiated MCF-7 cells, in dose dependent manner (**a**). Acetylcholinesterase (AChE) activity of conditioned media of the irradiated MCF-7 cells (**b**). AChE activity of irradiated conditioned media was significantly increased as compared to non-irradiated (control) conditioned media. One-way ANOVA with Tukey test was used. All values are shown as means ± SD; (3 biologically independent experiments). * P < 0.05, ** *P* < 0.01, *** P < 0.001

As shown in Fig. 3a, VEGF-A levels of CMs from irradiated MCF-7 cells significantly increased in 6 Gy group as compared to control group (*P*_*Control* vs. *6Gy group*_ *<* 0.05). VEGF-A levels of 8 Gy-CM group markedly elevated when compared with other groups (*P*_*8 Gy* vs. *Control, 2 Gy, and 4Gy groups*_ *<* 0.01). Similar to 8 Gy group, the protein level of VEGF-A in 10 Gy group significantly increased in comparison to other groups (*P*_*10 Gy* vs. *Control and 2 Gy groups*_ *<* 0.001; *P*_*10 Gy* vs.*4 Gy and 6 Gy groups*_ *<* 0.01)(Fig. 3a). Additionally, no difference was observed in VEGF-A content between 8 Gy and 10 Gy groups (*P* > 0.05).

### Ionizing radiation increased the acetylcholinesterase activity in conditioned media of MCF-7 cells

In addition to VEGF-A, we were interested to observe the secretion of extracellular vesicles in media after irradiation. Extracellular vesicles have been shown to act as paracrine factors [34]. Calculating the AChE activity, the extracellular vesicles content of CMs from MCF-7 cells was detected. We found a significant increase in the AChE activity of irradiated CMs as compared to non-irradiated control-CM (*P*_*Control* vs. *4Gy group*_ *<* 0.05; *P*_*Control* vs.*6 Gy and 8 Gy group*_ *<* 0.01; *P*_*Control* vs. *10 Gy group*_ *<* 0.001) (Fig. 3b). Compared to AChE activity in 2 Gy group (138.55 ± 4.73), AChE activity increased to 174.4 ± 8.34 and 200.33 ± 5.33 in 8 Gy and 10 Gy group (*P*_*2Gy group* vs. *8 Gy group*_ *<* 0.05; *P*_*2Gy group* vs. *10 Gy group*_ *<* 0.01).

In addition, the AChE activity rate of both 8 Gy and 10 Gy groups was greater than measured in 4 Gy group (*P* < 0.01). We also observed that ionizing radiation significantly increased the AChE activity in 10 Gy group, as compared to 6 Gy group (*P*_*6 Gy* group vs. *10 Gy group*_ < 0.01) (Fig. 3b). Collectively, the data show that ionizing radiation dose-dependently increased AChE activity in CMs, indicating that CM not only act through secreted VEGF-A but also through secreted vesicles.

### VEGFR-2, HSP-70, Ang-1, and Ang-2 expression was increased in endothelial cells incubated with irradiated conditioned media

**Fig. 4:**
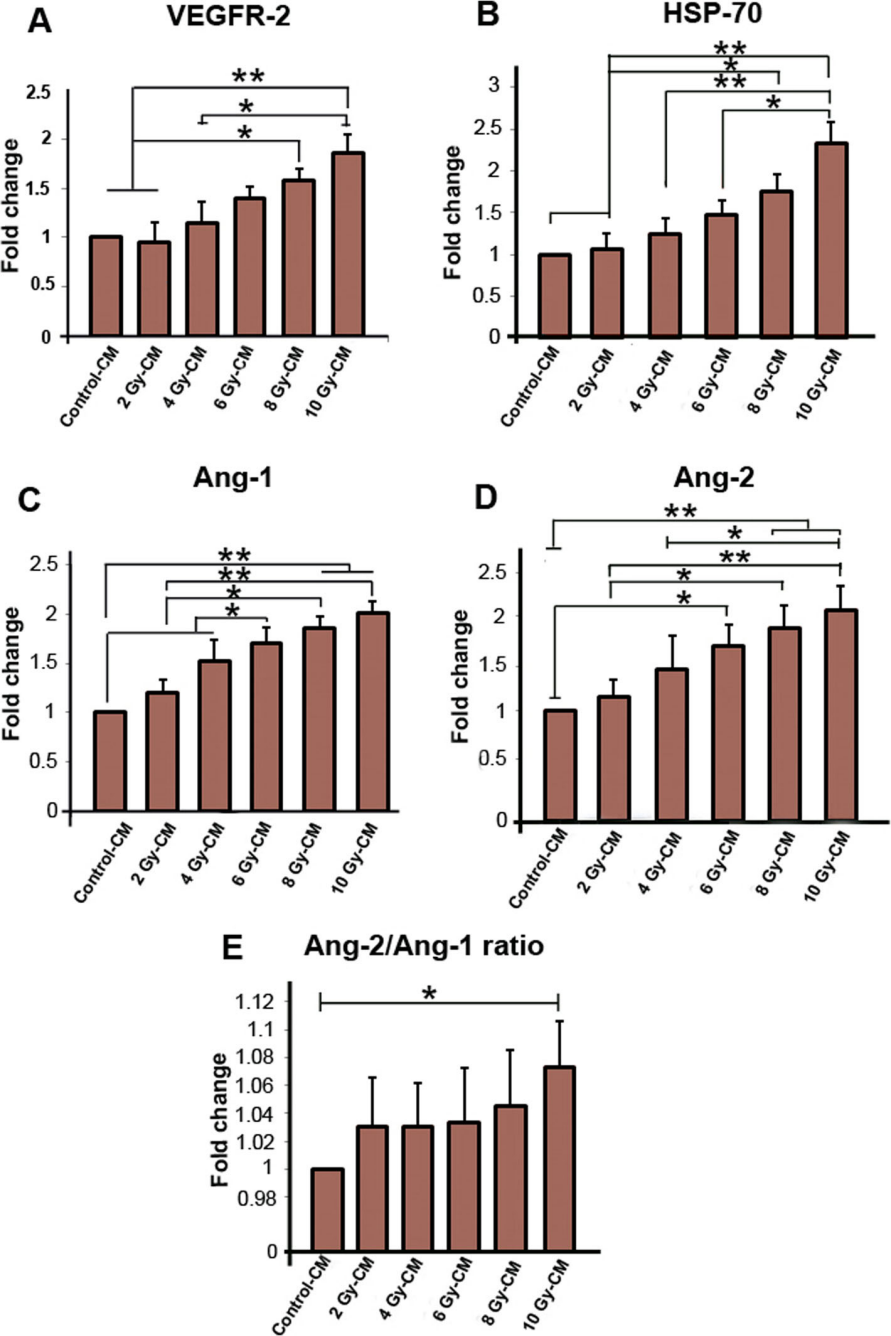
Real-time PCR analysis of mRNA expression for VEGFR-2, HSP-70, Ang-2, Ang-1 (**a**, **b**, **c**, **d**) and Ang-2/Ang-1 ratio (**e**) after incubation with irradiated conditioned media. Data were normalized against GAPDH expression. One-way ANOVA with Tukey test was used. All values are shown as means ± SD; (3 biologically independent experiments). * P < 0.05, ** *P* < 0.01

After confirming some of the secreted factors (e.g. secreted VEGF-A and secreted vesicles) in CM of irradiated MCF-7 cells, we were interested to observe the effect of these secreted factors for induction of gene expression in endothelial cells. The CMs of irradiated MCF-7 cells were incubated with HUVECs, and as molecular assessment, the mRNA levels of angiogenic genes including VEGFR-2, HSP-70, Ang-1, and Ang-2 were calculated by a real time-PCR. As shown in Fig. 4a, in comparison with either control-CM group or 2 Gy-CM group, a significant raise in the mRNA level of VEGFR-2 was observed in 8 Gy-CM and 10 Gy-CM groups (*P*_*Control-cm and 2Gy- CM* vs. *8Gy-CM group*_ *<* 0.05; *P*_*Control-cm and 2 Gy-cm* vs.*10 Gy-CM group*_ *<* 0.01). Conditioned media from 10 Gy markedly induced the expression of VEGFR-2 as compared to 6 Gy-CM group (*P*_*6 Gy-cm* vs.*10 Gy-CM group*_ *<* 0.05).

Furthermore, the transcript levels of HSP-70 enhanced in 8 Gy-CM and 10 Gy-CM groups as compared to both control-CM and 2 Gy-CM groups (*P*_*Control-cm and 2Gy- CM* vs. *8Gy-CM group*_ *<* 0.05; *P*_*Control-cm and 2 Gy-cm* vs.*10 Gy-CM group*_ *<* 0.01) (Fig. 4b). We found that the mRNA levels of HSP-70 significantly amplified in 10 Gy-CM group when compared to 4 Gy-CM and 6 Gy-CM groups (*P* < 0.01 and *P* < 0.05; respectively).

Additionally, we measured the expression of Ang-1 and Ang-2 genes, the angiogenesis regulator genes, in HUVEC over a period of 48 h exposure. Data showed that in comparison with control-CM group the mRNA levels of Ang-1 were increased in HUVECs incubated with irradiated CMs (*P*_*Control-cm* vs. *4 Gy-CM and 6 Gy-CM group*_ < 0.05; *P*_*Control-cm* vs. *8Gy-CM and 10 Gy-CM group*_ *<* 0.01) (Fig. 4c). In addition, the expression of Ang-1 gene significantly increased in 8 Gy-CM group (1.86 ± 0.1 fold) and 10 Gy-CM (2.02 ± 0.109 fold) as compared to 2 Gy-CM group (1.2 ± 0.14 fold; *P*_*Control-cm* vs. *8Gy-CM group*_ *<* 0.01; *P*_*Control-cm* vs. *10 Gy-CM group*_ *<* 0.01).

Similarly, apparent from Fig. 4d, it was found that upon incubation with irradiated CMs the mRNA levels of Ang-2 were markedly enhanced (*P*_*Control-cm* vs.*6 Gy-CM group*_ < 0.05; *P*_*Control-cm* vs. *8Gy-CM and 10 Gy-CM groups*_ *<* 0.01). Compared to 2Gy-CM, the mRNA levels of Ang-2 were increased in 8 Gy-CM and 10 Gy-CM groups (*P*_*2Gy-cm* vs.*8 Gy-CM group*_ < 0.05; *P*_*2Gy-cm* vs. *10 Gy-CM groups*_ *<* 0.01). Additionally, we observed a significant increase in the Ang-2 transcript levels of 10 Gy-CM (2.17 ± 0.18 fold) vs. those of 4 Gy-CM group (1.58 ± 0.251; *P* < 0.05) (Fig. 4d). Determination of Ang-2/Ang-1 ratio may correspond to angiogenic switch. Interestingly despite an increasing trend in Ang-2/Ang-1 ratio, we found only a significant increase in 10 Gy-Cm group (1.073 ± 0.033) as compared to control-CM group (P < 0.05) (Fig. 4e). Collectively, the results showed that the exposure of conditioned media from irradiated MCF-7 cells upregulated the angiogenic genes in HUVECs. This indicates that, through secretory factors, the irradiated cells relay their bystander effects to endothelial cells for the induction of the expression of angiogenic genes.

### Irradiated conditioned media enhanced the capillary-like networks in three-dimensional culture

**Fig. 5:**
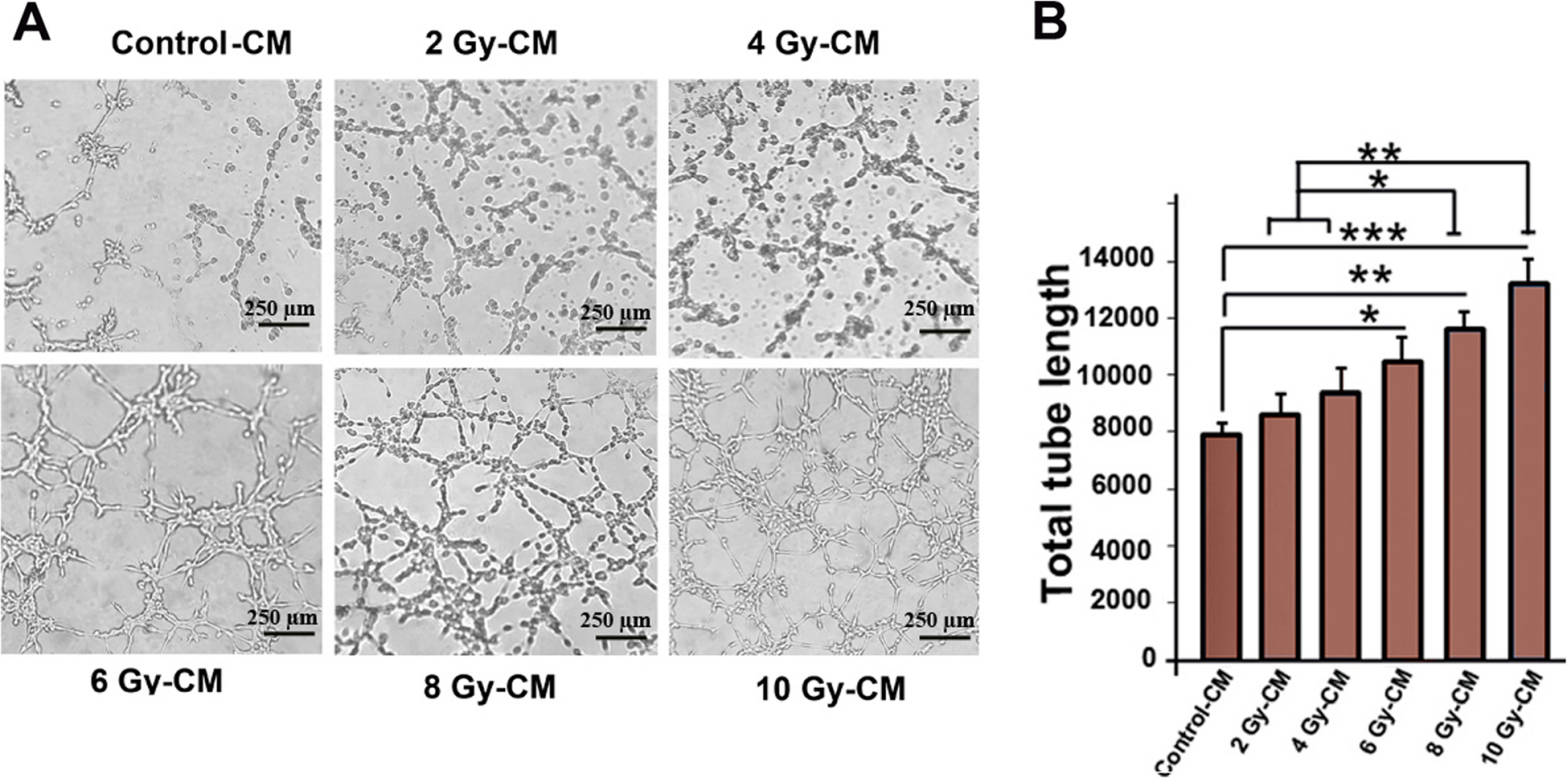
Representative images of Matrigel tube formation of HUVECs pre-incubated with conditioned media from MCF-7 cells. Comparison of total length of tube showed an increased tubulogenesis ability of HUVECs after receiving irradiated conditioned media (**a**, **b**). (Scale bar: 250 μm). All values are shown as means ± SD; (3 biologically independent experiments). * P < 0.05, ** *P* < 0.01, *** P < 0.001

After molecular analysis, we aimed to examine the phenotypic effects of irradiated secretory factors on endothelial cells, and thus examined the tube/capillary formation in HUVECs. Tube-formation assay is one of the simple, yet well-established in vitro angiogenesis assays based on the ability of endothelial cells to form three-dimensional capillary-like tubular structures. Our data show that when pretreated HUVECs were cultured on Matrigel, the capillary like networks increased in a dose-dependent manner in cells pre-treated irradiated CMs (Fig. 5a, b). Compared to control group, the value of total tube length was significantly increased in cells cultured with 6 Gy, 8 Gy, and 10 Gy irradiated CMs (*P*_*Control-cm* vs.*10 Gy-CM group*_ < 0.05; *P*_*Control-cm* vs. *8Gy-CM group*_ *<* 0.01; *P*_*Control-cm* vs.*10 Gy-CM group*_ *<* 0.001). We found that the HUVECs exposed to 8 Gy and 10 Gy CMs induced the profound tube formation capability in comparison to 2 Gy and 10 Gy CMs (*P*_*2Gy-cm and 4 Gy-CM* vs. *8 Gy-CM group*_ < 0.05*; P*_*2 Gy-cm and 4 Gy-CM* vs.*10 Gy-CM group*_ < 0.01) (Fig. 5a, b). We propose that these angiogenic effects could be mediated both by transfer of extrinsic secreted factors (VEGF-A, and extracellular vesicles) to HUVECs as well as by induction of intrinsic gene expression in HUVECs.

### Irradiated conditioned media enhanced the CD34 positive HUVECs

**Fig. 6:**
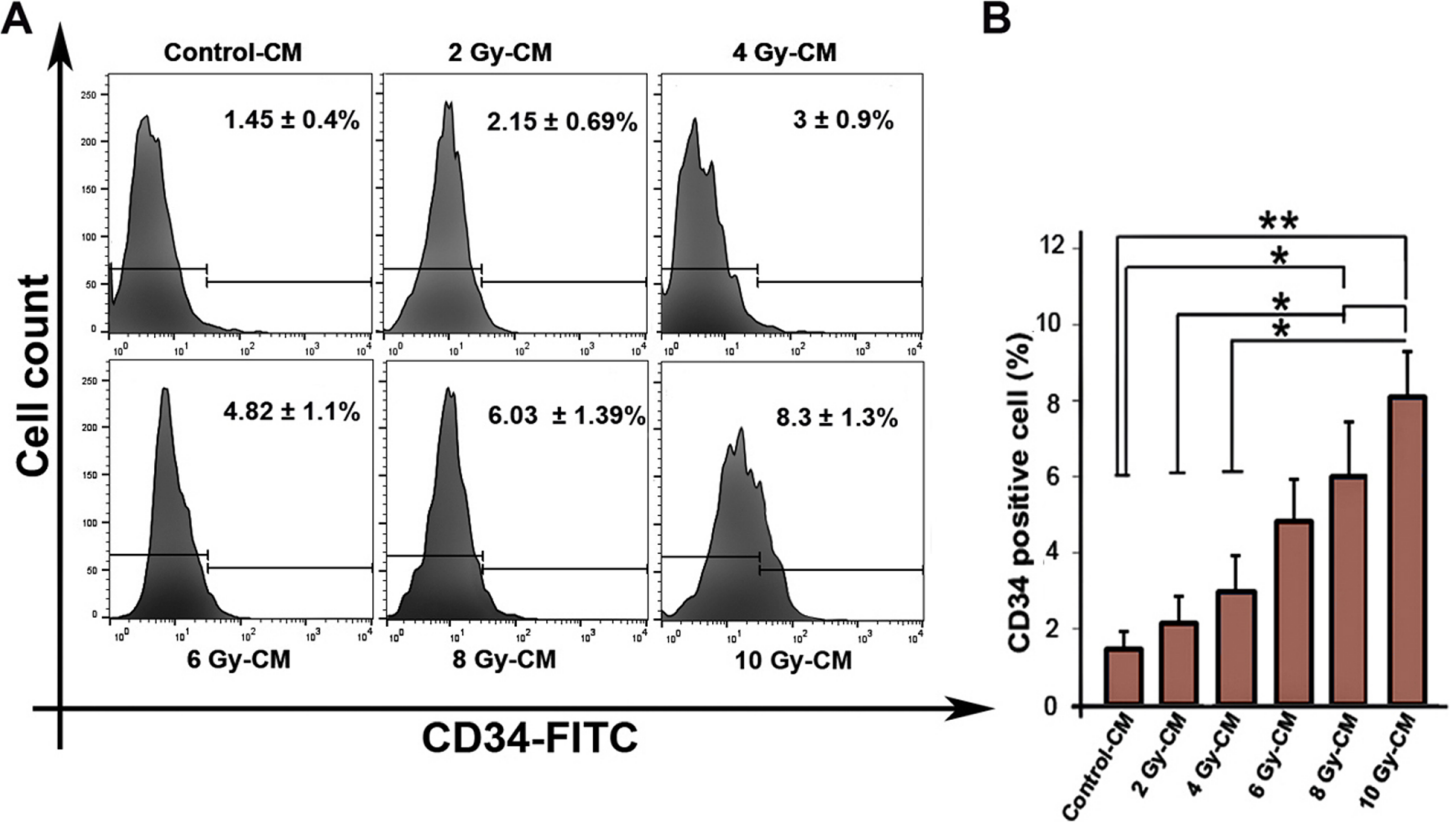
Flow cytometry analysis of CD34 marker. Data shows the increasing percentage of CD34 positive HUVECs exposed to irradiated conditioned media over 48 h (**a**, **b**). One-way ANOVA with Tukey test was used. All values are shown as means ± SD; (3 biologically independent experiments). * P < 0.05, ** *P* < 0.01

CD34 is a marker of angiogenic endothelial tip cells, where tip cell phenotype have been shown to exhibit biological functions related to angiogenesis [35]. We studied the effect of irradiated CMs on the differentiation potency of HUVECs into CD34 positive cells. Through the incubation with irradiated CMs, the percentage of CD34 positive cells was increased in comparison to control group (*P*_*Control-cm* vs. *8Gy-CM group*_ *<* 0.05; *P*_*Control-cm* vs.*10 Gy-CM group*_ *<* 0.01) (Fig. 6a, b). Compared to 2 Gy-CM group, cells incubation with 8 Gy and 10 Gy conditioned media was found to increase the percentage of CD34 positive endothelial cells up to 6.03 ± 1.39% and 8.3 ± 1.3% respectively (*P* < 0.05). Additionally, an increased level of CD34 positive cells was observed in 10 Gy-CM group as compared to 4 Gy-CM group (*P* < 0.05)(Fig. 6a, b).

## Discussion

Radiotherapy, applying ionizing radiation to eradicate tumor mass, represents clinical concerns such as non-targeting effects in patients who received ionizing radiation [36]. In recent years, the non-targeting effects irradiation (i.e. radiation-induced bystander effects) have been investigated to evaluate the cell responses in non-irradiated cells [11, 13]. Bystander effects of irradiated cells contribute to influence low irradiated and non-irradiated cells including tumor and endothelial cells [37, 38].

We provide in vitro data that the conditioned media from irradiated MCF-7 cells act as mediator induction of gene expression and promote angiogenesis in endothelial cells. We found that number of migrated HUVECs toward irradiated CMs is amplified in a dose-dependent manner. This is in good agreement with a study that CMs from X-rayed C6 glioma cells could induce the endothelial cells’ migration in vitro [30] and also with Arscott et al. work that the extracellular vesicles derived from CMs of irradiated glioblastoma cells could potentially enhance the migration of non-irradiated cells [39].

In addition, wound healing assay further confirms the migration ability of HUVECs in that, the percentage of wound healing rate dose-dependently increased at the endpoint of exposure. More recently, it was demonstrated that the CMs collected from irradiated human lung cancer cells enhanced the non-irradiated cells’ migration rate [40]. These results highlight the induction of migration responses of HUVECs against irradiated CMs, however the underlying mechanisms largely remain unknown. To investigate the possible mechanisms, dealing with the chemotaxis/migration behavior of HUVECs, we measured the protein content of VEGF-A in CMs of MCF-7 cells. It is important to note that the amount of secreted VEGF-A factor in the CMs of irradiated cells was elevated according to increase in X-ray doses. This finding could be valuable due to the key roles of VEGF-A in angiogenesis. It seems that secreted VEGF-A in CMs acts as chemoattractant, so that the migration and wound healing rate of HUVECs increased simultaneously with raise in VEGF-A content of CMs. Our results share a number of similarities with previous findings that ionizing radiation induces the VEGF-A production in irradiated cells, which could be quantified in the supernatants of irradiated cells [41, 42]. Furthermore, our data show that the activity of acetylcholinesterase, an enzyme linked to extracellular vesicles, is increased in CMs in a dose dependent manner. This implies the elevated level of extracellular vesicles in CMs [43], and substantiates previous findings in the literature [44, 45]. In our opinion, extracellular vesicles derived from irradiated cells by transferring different biomolecules, mediate the radiation-induced bystander effects in recipient cells [46, 47], and contribute to induce cell migration [39].

Keeping in view the secretion of paracrine factors in CM by irradiated cells and their co-incubation with HUVECs, it may be assumed that HUVECs respond against ionizing radiation damage by a proangiogenic system. Further tests carried out with HUVECs confirmed that the mRNA levels of VEGFR-2, the VEGF receptor, were increased in irradiated CMs in a dose-dependent manner after 48 h incubation.

Up-regulation of VEGFR-2 was concurrent with enhanced cell migration and tubulogenesis in HUVECs. Of note, VEGF-A/VEGFR-2 signaling pathway contributes to endothelial cells’ migration and promotes angiogenesis [48]. Regarding enhanced VEGF-A content in irradiated CMs and increased VEGFR-2 transcripts in HUVECs, it can thus be proposed that the downstream signaling of VEGF-A /VEGFR-2 axis may participate to enhance wound healing and migration rate. Moreover, we found that the mRNA levels of HSP-70 dose-dependently increased. It was suggested that the expressions of HSP-70 are elevated in response to environmental stress [49]. Besides, recently, the pivotal role of HSP-70 in angiogenesis has been reported [50, 51]. It was found that HSP-70 induces migration response of lymphocytes [52], whereas Kasioumi et al. found that down-regulation of HSP-70 causes a profound migration rate in cells [53]. To our knowledge, this is a preliminary report and further investigation is needed to elucidate the underlying mechanism related to HSP-70 pathway.

Furthermore, our study shows the up-regulation of Ang-1 and Ang-2 factors in HUVECs co-cultured with irradiated CMs. Ang-2 and -1 participate in the dynamic of angiogenesis and vascular network [54, 55]. We also calculated the Ang-2/Ang-1 ratio in cells. In this regard, an increasing trend in Ang-2/Ang-1 ratio was observed; however, a significant difference between control-CM and 10 Gy-CM groups was obtained, confirming the augmented angiogenesis in 10 Gy-CM group (Fig. 4e). Seemingly, this result is arising from 10 Gy irradiated CM, which could activate angiopoietin system in HUVECs against CM’s ingredients. The increased signature of Ang-2, Ang-1, Ang-2/Ang-1 ratio and VEGF-A/VEGFR-2 axis may participate in high ability of HUVECs in increasing angiogenesis [17, 56]. Data showed that expression of angiogenic genes up-regulated in endothelial cells co-cultured with CMs from irradiated MCF-7 cells. This indicates that the secreted factors in CMs against irradiation play key roles in the communication with endothelial cells, which may influence expression system in recipient HUVECs.

The induction of angiogenic switch in HUVECs indicates the participation of bystander effects in amplified angiogenic status in vitro. In an effort to have additional angiogenesis assessment, in agreement with Liu and co-workers study [57], we found that the irradiated CMs promote tubulogenesis in the 3D system. To shed light onto possible underlying mechanism, we monitored the percentage of CD34 positive cells in all groups. Our findings showed that CD34 positive cells were increased in a dose-dependent relationship. In the angiogenesis process, CD34 marker expresses in a small number of endothelial cells and these cells form cellular protrusions known as tip cells which are a hallmark of development of angiogenesis [35]. On combining this result with tube formation assay, we hypothesized that the augmented in vitro vascular network may partially correlate with percentage of CD34 positive cells. It has been reported that CMs from irradiated cells potentially contain various growth factors [58, 59] that may contribute to induce angiogenesis in HUVECs. In line with our results, the augmented VEGF-A proteins and EVs in irradiated CMs as well as the high mRNA levels of angiogenic factors in HUVECs may explain the augmented tubulogenesis potential of HUVECs.

**Fig. 7:**
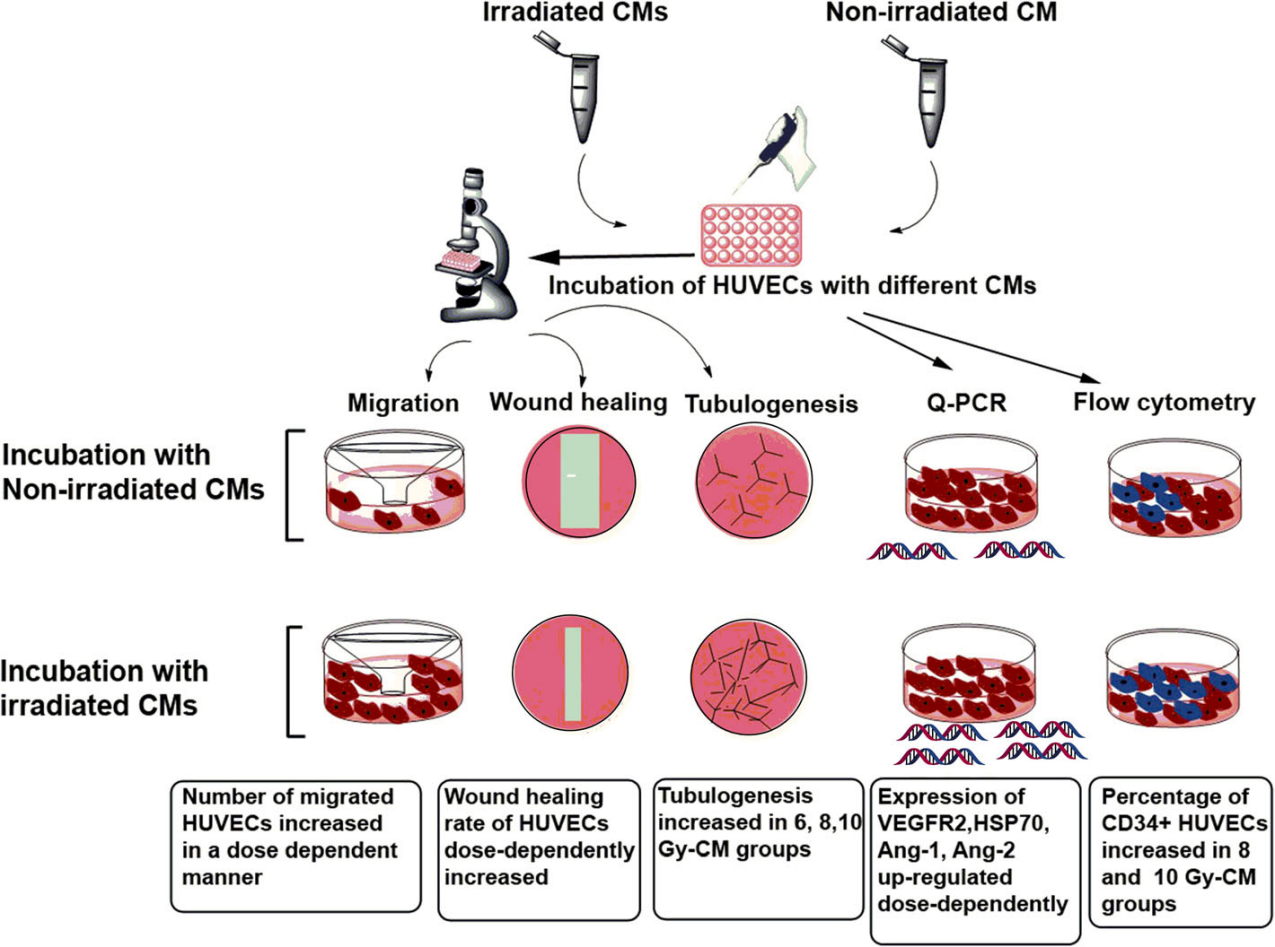
A schematic illustration showing the biological effects of irradiated conditioned media on migration, wound healing rate, tubulogenesis, expression of VEGFR2, HSP70, Ang-1, and Ang-2 genes, and percentage of CD34+ HUVECs. CM: conditioned media; Q-PCR: Quantitative PCR

Collectively, our data indicate that the migration, induction of gene expression and angiogenic responses in endothelial cells against conditioned media from irradiated MCF-7 cells, could be due to transfer of overexpressed secreted VEGF-A and other secreted factors from irradiated cells to endothelial cells, which may act as chemoattractant or/and induce the expression of intrinsic genes (VEGFR-2, HSP-70, Ang-2, and Ang-1) in endothelial cells, which enhance their angiogenic and migration ability (Fig. 7).

## Conclusion

We showed that conditioned media from MCF-7 cells, which was exposed to various doses of X-ray (2, 4, 6, 8, and 10 Gy) were able to induce gene expression and promote HUVECs angiogenic potential over 48 h incubation. By further studying the angiogenic responses of HUVECs, we may address the underlying mechanism of paracrine intercellular communication within irradiated tumor tissue and surrounding cells during radiotherapy. These features may be valuable in foresight into bystander effects of radiotherapy in tissues with secondary tumors to monitor angiogenesis.

## Data Availability

Not applicable.

## Abbreviations

AChE: Acetylcholinesterase
BEs: Bystander Effects
bFGF: Basic fibroblast growth factor
CM: Conditioned media
EG: Epidermal growth factor
ELISA: Enzyme-linked immunosorbent assay
EVs: Extracellular vesicles
HUVECs: Human umbilical vein endothelial cells
MMPs: Matrix metalloproteinase
NTE: Non-targeting Effects
TnTs: Tunneling nanotubes
uPA: urokinase-type plasminogen activator
VEGF: Vascular endothelial growth factor
